# The Physical Adsorption of Gelatinized Starch with Tannic Acid Decreases the Inhibitory Activity of the Polyphenol against α-Amylase

**DOI:** 10.3390/foods10061233

**Published:** 2021-05-28

**Authors:** Yueyi Wang, Shuangshuang Li, Fangting Bai, Junwei Cao, Lijun Sun

**Affiliations:** College of Food Science and Engineering, Northwest A & F University, Xianyang 712100, China; yueyiwang2021@163.com (Y.W.); Xiaoxiaoyu1499@163.com (S.L.); b3519526@163.com (F.B.); cjw20170725@163.com (J.C.)

**Keywords:** α-amylase inhibition, tannic acid, mixing order, binding interactions, adsorption

## Abstract

The effects of mixing orders of tannic acid (TA), starch, and α-amylase on the enzyme inhibition of TA were studied, including mixing TA with α-amylase before starch addition (order 1), mixing TA with pre-gelatinized starch before α-amylase addition (order 2) and co-gelatinizing TA with starch before α-amylase addition (order 3). It was found that the enzyme inhibition was always highest for order 1 because TA could bind with the enzyme active site thoroughly before digestion occurred. Both order 2 and 3 reduced α-amylase inhibition through decreasing binding of TA with the enzyme, which resulted from the non-covalent physical adsorption of TA with gelatinized starch. Interestingly, at low TA concentration, α-amylase inhibition for order 2 was higher than order 3, while at high TA concentration, the inhibition was shown with the opposite trend, which arose from the difference in the adsorption property between the pre-gelatinized and co-gelatinized starch at the corresponding TA concentrations. Moreover, both the crystalline structures and apparent morphology of starch were not significantly altered by TA addition for order 2 and 3. Conclusively, although a polyphenol has an acceptable inhibitory activity in vitro, the actual effect may not reach the expected one when taking processing procedures into account.

## 1. Introduction

Postprandial hyperglycemia is an important factor that causes disturbance of glucose metabolisms, like type II diabetes. Starchy foods are the main source of carbohydrates for human beings, the digestion of which decides the changes of postprandial blood sugar level to a large extent. α-Amylase is a key carbohydrate-hydrolyzing enzyme that initially catalyzes starchy components, producing reducing sugars, such as maltose, maltotriose, maltooligosaccharides, etc., and these reducing sugars are further hydrolyzed by α-glucosidase to glucose that is finally absorbed by enterocytes in the small intestine [1]. Therefore, inhibiting the activity of α-amylase by introducing exogenous enzyme inhibitors has been considered effective in controlling blood glucose level after meals through delaying starch digestion [2].

Natural polyphenols or phenolic extracts from plant foods have been reported to develop the inhibitory activity against α-amylase, retarding starch digestion both in vitro and in vivo [3]. Notably, α-amylase inhibition of a phenolic compound results from non-covalent binding interactions between them, mainly including hydrogen bondings and π-π conjugations [4,5]. This way, the factors that affect polyphenol–amylase binding interactions are considered to cause the changes in the inhibitory activity of polyphenols; for example, there is a structure–activity relationship for flavonoids regarding α-amylase inhibition as the difference in flavonoid structures results in the difference in binding affinity of the polyphenols to the enzyme [5,6]. Soluble polysaccharides (oat β-glucan and wheat arabinoxylan) have been reported to decrease the inhibitory activity of tea polyphenols against α-amylase, because the polysaccharides could also bind with the polyphenols, decreasing the binding interactions between tea polyphenols and the enzyme [7,8]. More factors that influence polyphenol–amylase binding and thus influence the inhibiting effect need to be explored to give a better understanding for further developing the inhibitory activity of a phenolic compound.

Usually, to characterize the inhibiting effect of a polyphenol against α-amylase, the polyphenol is mixed with the enzyme firstly, followed by addition of substrates (e.g., starch) [9,10]. This way, polyphenols can interact with α-amylase thoroughly before the substrate digestion occurs. Therefore, to develop the inhibitory activity of a polyphenol in vivo, it is necessary to ensure the polyphenol contact and interact with α-amylase before starchy foods reach at small intestine where the enzyme plays the catalyzing role. However, in the actual situation, polyphenols are not always necessarily ingested before starch components. Moreover, the starch substrates (amylose and amylopectin) are biomacromolecules that may also bind/absorb with polyphenol biomicromolecules [11,12,13], especially when the two compounds fully contact with each other. To our knowledge, whether the binding of a polyphenol with starch would affect the binding of the polyphenol with α-amylase and thus affect the inhibition effect are still unclear. Therefore, the enzyme inhibition under different mixing orders of enzyme, starch, and polyphenol needs to be shed light on. Tannic acid (TA, one kind of tannin component, composed of 10 galloyl moieties and 1 glucosyl base) widely exists in vegetables and fruits such as persimmon, grape peel, pomegranate peel, etc. It has been suggested as an effective inhibitor of α-amylase that can bind with the active site of the enzyme [14,15]. Hence, it is a good inhibitor model compound in the study of inhibition property. Therefore, in this study the effects of three common mixing orders of TA, starch, and α-amylase (mixing TA with α-amylase before starch addition; mixing TA with pre-gelatinized starch before α-amylase addition; co-gelatinizing TA with starch before α-amylase addition) on the enzyme inhibition of TA are explored, through which how the adsorption of TA with starch affects the binding of TA with α-amylase is also illustrated.

## 2. Materials and Methods

### 2.1. Materials and Reagents

Tannic acid (TA), porcine pancreatic α-amylase (10080, 50 U/mg), and phosphate- buffered saline (PBS) tablets were purchased from Sigma-Aldrich Co. (St. Louis, MO, USA). EnzCheck^TM^ Ultra Amylase Assay Kit was purchased from Life Technologies Co. (Carlsbad, CA, USA). Maize starch and *p*-hydroxybenzoic acid hydrazide (PAHBAH, CAS No. 5351-23-5) were obtained from Yuanye Biotech Co. (Shanghai, China). Other reagents were of analytical grade.

### 2.2. Three Mixing Orders of Tannic Acid, α-Amylase, and Starch

The three mixing procedures applied in this study (Figure S1) were as follows: (1) TA (dissolved in PBS buffer) was mixed with α-amylase (in PBS buffer) and incubated at 4 °C for 15 min. After that, the gelatinized starch (that was cooked at 90 °C for 20 min) was added in to the mixture of TA and α-amylase to start the reaction at 37 °C; (2) TA was mixed with gelatinized starch at 37 °C for 15 min, and then α-amylase was added to the mixture of TA and gelatinized starch to start the reaction at 37 °C; (3) TA was mixed with raw starch, followed by co-gelatinization at 90 °C for 20 min. After that, α-amylase was added to the co-gelatinized TA-starch to start the reaction at 37 °C.

### 2.3. α-Amylase Inhibition of TA

#### 2.3.1. α-Amylase Inhibition Characterized by Starch Digestion

The inhibition effects of TA for the three mixing procedures were initially characterized by determination of the initial reaction velocity of starch digestion in the absence and presence of TA [16]. The contents of reducing sugars (maltose equivalents) were determined by the PAHBAH method [16], and inhibition (%) was calculated by the following Equation (1):

(1) $$\mathrm{Inhibition}\left(\%\right)=\left(1-\frac{v}{v_{0}}\right)\times 100$$

where *v* and *v*_0_ are the initial reaction velocity of starch digestion in the presence and absence of TA, respectively, which were obtained from the slopes of the linear correlations between the reducing sugar contents and digestion time.

#### 2.3.2. Time Course of Starch Digestion

To observe the enzyme inhibition effect during the whole process of substrate digestion, a time course of starch digestion in the absence and presence of TA was recorded for the three mixing procedures. Specifically, 6 mL of 15 mg/mL gelatinized starch, 100 μL of 25 mg/mL TA, and 100 μL of 1 mg/mL α-amylase (or the equivalent amounts of them) were prepared for digestion according to the three procedures introduced above. During the time course of starch digestion, the contents of reducing sugar produced at individual time interval were determined by the PAHBAH method [16]. Then, the correlations between the digested starch fraction (conversion coefficient of maltose to starch is 324/342) and digestion time were analyzed by the first-order Equation (2) as follows [17]:

(2) $$C_{t}=C_{\infty }\left(1-e^{-kt}\right)$$

where *t* is the digestion time; *C_t_* is the fraction of digested starch at digestion time *t*; *C*_∞_ is the fraction of digested starch at the end point of the reaction; and *k* is the digestion rate constant. To obtain the value of *k*, Equation (2) can be transformed into a logarithm of slope (LOS) plot in which there is a linear correlation between *ln*(*dC_t_*/*dt*) and *k* as follows [18]:

(3) $$ln\left(\frac{dC_{t}}{dt}\right)=-kt+ln\left(C_{\infty }k\right)$$

For the starch fractions digested at a single rate, the LOS plot is linear, while others may have multiple distinct linear phases. In this case, the whole starch digestion can be expressed as a piecewise function as follows [19]:

(4) $$Ct=\{\begin{array}{c} C_{1}+C_{1\infty }\left(1-e^{-k_{1}t}\right),\;0\leq t\leq t_{1}\\ C_{2}+C_{2\infty }\left(1-e^{-k_{2}t}\right),\;t_{1}\leq t\leq t_{2}\\ \ldots \\ C_{n}+C_{n\infty }\left(1-e^{-k_{n}t}\right),\;t_{n-1}\leq t\leq t_{n} \end{array}$$

where *n* depends on the number of phases. In each phase, *k_n_* and *C_n_*_∞_ represent the corresponding starch digestion rate constant and fraction of digested starch at the end point of each reaction phase; *C_n_* is the starting fraction of digested starch in each phase; *t_n_* is the terminal time of each phase.

### 2.4. Mechanism in α-Amylase Inhibition of TA

#### 2.4.1. IC_50_ Value

IC_50_ value could reasonably reflect the inhibitory activity of a phenolic compound. To obtain this value based on the inhibition (%) ranging from low to high values at a series of available TA concentrations, an EnzCheck^TM^ Ultra Amylase Assay Kit was applied for order 1. The enzymic inhibition (*I*) was calculated according to Equation (1) and the IC_50_ value of TA was obtained using Equation (5) as follows [20]:

(5) $$I=I_{max}\left(1-\frac{IC_{50}}{\left[I\right]+IC_{50}}\right)$$

where [*I*] is the TA concentrations; *I* is α-amylase inhibition at each TA concentration; *I_max_* is the maximum inhibition.

#### 2.4.2. Inhibition Kinetics

The inhibition kinetics of TA for order 1 and 2 were studied by applying Dixon and Cornish-Bowden plots according to the previously reported methods [16]. The competitive inhibition constant *K_ic_* and the uncompetitive inhibition constant *K_iu_* were obtained from the Dixon (6) and Cornish-Bowden (7) equations respectively as follows [21,22]:

(6) $$v=\frac{V_{max}a}{K_{m}\left(1+\frac{i}{K_{ic}}\right)+a}$$

(7) $$\frac{v}{a}=\frac{V_{max}}{K_{m}\left(1+\frac{i}{K_{ic}}\right)+a\left(1+\frac{i}{K_{iu}}\right)}$$

where *v* is the initial reaction velocity of starch digestion; *V_max_* is the maximum initial reaction velocity; *a* is the starch concentration; *K_m_* is the Michaelis constant; *i* is the TA concentration.

#### 2.4.3. Fluorescence Quenching

The fluorescence spectra of α-amylase in the absence and presence of TA with various concentrations were determined by using a spectrofluorometer (LS55, Perkin Elmer, Waltham, MA, USA). The fluorescence quenching constant, *K_FQ_*, was calculated from the Stern-Volmer Equation (8) as follows [23]:

(8) $$\frac{F_{0}}{F}=1+k_{q}\tau_{0}\left[Q\right]=1+K_{FQ}\left[Q\right]$$

where *F*_0_ and *F* are the fluorescence intensity in the absence and presence of TA, respectively; *k_q_* is the bimolecular quenching constant; *τ*_0_ is the lifetime of the fluorophore, and for α-amylase this value is 2.97 ns; [*Q*] is the quencher (TA) concentration.

### 2.5. Adsorption of TA with Starch

It seems inconvenient to measure the adsorption property of TA onto gelatinized starch directly. Thus, a dialysis method was introduced in this study for two preparation procedures including (1) mixing TA with pre-gelatinized starch and (2) co-gelatinizing starch with TA. Both the gelatinization processes were conducted at 90 °C for 20 min. The final concentration of starch in the gelatinized system was 10 mg/mL, and the TA concentrations were in a series of 2, 4, 6, and 8 mg/mL. The respective gelatinized TA-starch solutions for two procedures were placed into a dialysis bag with the membrane cut-off molecular weight of 7 kD (TA can pass through the dialysis membrane but the starch macromolecules cannot). To start the dialysis process, the dialysis bag was put into a beaker containing PBS buffer, and the TA concentrations outside the dialysis bag at individual time points were determined by the Folin–Ciocalteu method with a standard curve of absorbance value against TA concentration. The dialysis of TA in the absence of starch was used as the control. Then, the binding capacity (*B*, the adsorption amount of TA per mass of starch) of starch was calculated according to Equation (9) as follows:

(9) $$B=\frac{(C_{0}-C_{t})V}{m}$$

where *C*_0_ and *C_t_* are the respective TA concentrations outside the dialysis bag of control and TA-starch samples at a dialysis time *t*; *V* is the solution volume in the beaker; *m* is the mass of gelatinized starch in the dialysis bag.

To describe the free diffusion property of TA, the first-order pharmacokinetics Equation (10) was applied to analyze the correlation between the TA concentration outside the bag and dialysis time as follows [24,25]:

(10) $$C_{t}=C_{\infty }\left(1-e^{-k_{t}t}\right)$$

where *C_t_* is the TA concentration outside the dialysis bag at time *t*; *C*_∞_ is the TA concentration outside the dialysis bag at the dialysis equilibrium; *k_t_* is the transport rate constant of TA. To obtain the value of *k_t_*, the equation (10) can be transformed into a logarithm of slope plot (11) in which there is a linear correlation between *ln*(*dC_t_*/*dt*) and *k_t_* as follows:

(11) $$ln\left(\frac{dC_{t}}{dt}\right)=-kt+ln\left(C_{\infty }k_{t}\right)$$

### 2.6. Binding Interactions between TA and Starch

To characterize the binding interactions between TA and starch, the gelatinized TA-starch complexes were prepared based on two procedures including (1) mixing TA with pre-gelatinized starch and (2) co-gelatinizing starch with TA. For procedure (1), 40 mL of 10 mg/mL pre-gelatinized starch was mixed with 1 mL of TA with a series of concentrations (10–60 mg/mL), while for procedure (2), 40 mL of 10 mg/mL raw starch suspension was mixed with 1 mL of TA with a series of concentrations (10–60 mg/mL), followed by co-gelatinization at 90 ℃ for 20 min. Both the TA-starch gelatinized complexes were then lyophilized, ground, and sieved (200-mesh). The obtained powder was analyzed by FTIR, XRD, and SEM to characterize the changes in molecular structures and apparent morphology of starch caused by TA addition, from which the binding interactions between them may be also reflected.

#### 2.6.1. FTIR Analysis

Before FTIR analysis, all the samples were dehydrated at 45 °C for 3 h to minimize the impact of the residual water component. Then, the samples (~5 mg) were mixed with KBr (1:100) and pressed into a semitransparent pellet. The FTIR spectra were recorded by use of an IR spectrometer (Tensor27, Bruker, Billerica, MA, USA) within the frequency range of 4000 to 400 cm^−1^, and the sample was scanned 32 times with the resolution of 4 cm^−1^.

#### 2.6.2. X-ray Diffraction

X-ray diffraction (XRD) was conducted to explore the crystalline structure of the TA-starch complex samples. After drying the samples at 45 °C for 3 h, they were further ground finely and scanned from 10° to 60° at a step size of 0.02 and a time interval of 0.5 s per step by using a Powder X-ray Diffractometer (D8 Advance, Germany) equipped with a CuKα lamp and a nickel filter.

#### 2.6.3. SEM Observation

For SEM observation, the lyophilized starch samples were fractured and sprayed with gold powders at the surfaces, followed by being imaged by use of an Environmental Scanning Electron Microscope (ESEM, Quanta 200, FEI, Hillsborough, OR, USA) with a magnification of 4000×. The operation was performed in a high vacuum mode and 20 kV.

### 2.7. Statistical Analysis

One-way analysis of variance (ANOVA) by Graphpad Prism 6 software was applied to analyze the significant difference between the data obtained. When *p* < 0.05, the data is considered as statistically significant and thus marked with different superscripts.

## 3. Results

### 3.1. α-Amylase Inhibition of TA

**Figure 1:**
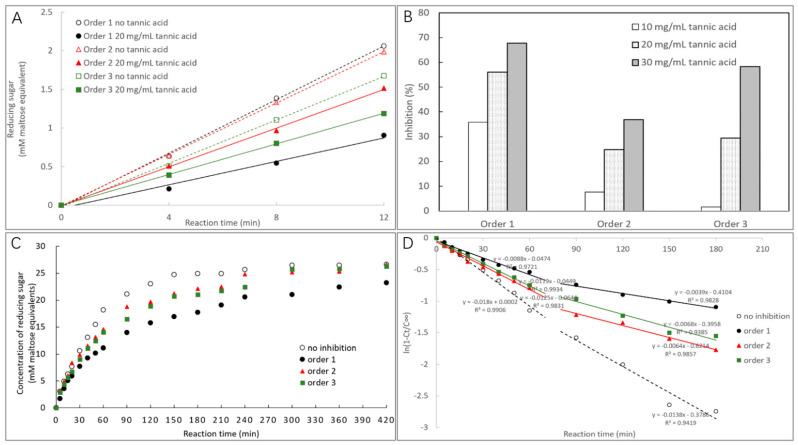
The contents of reducing sugars (maltose equivalents) produced along with starch digestion at a time interval of 4 min in the absence and presence of TA for three mixing procedures, and the initial reaction velocity was obtained from the slope of plot of reducing sugar contents against digestion time (**A**). Based on this, the enzyme inhibition of TA for three mixing procedures at the low (10 mg/mL) and high (20 and 30 mg/mL) TA concentrations were obtained (**B**). The time course of starch digestion in the absence and presence of TA for three mixing procedures (**C**). The logarithm of slope (LOS) analysis for the fraction of digested starch along with digestion time (**D**), from which the digestion rate constants *k* in each digestion phases were obtained.

The activity of α-amylase in the presence and absence of TA for the different mixing orders of TA, starch, and α-amylase were indicated by the initial reaction velocity (Figure 1A). Based on this, the inhibition at different TA concentrations for the respective mixing orders were obtained. As shown in Figure 1B, the inhibition effect of TA was the highest for order 1 at each TA concentration. Interestingly, at the low TA concentration (10 mg/mL), the inhibition for order 2 was higher than order 3, while at the high TA concentrations (20 and 30 mg/mL), the inhibition for order 3 was higher than order 2 (Figure 1B and Table 1). Then, to characterize the inhibition (retarding) effect on starch digestion process and the digestibility property, the time course of starch digestion in the presence and absence of TA were obtained (Figure 1C). Order 1 was always found with the strongest retarding effect on starch digestion during 7 h (Figure 1C). Notably, the mass ratio of TA to starch (1:36) in the time course study fell in between the low (1:40, mass ratio of TA/starch) and high (1:20) TA concentrations in the enzyme inhibition study above (Figure 1B), which accorded with the fact that the retarding effect on starch digestion for order 3 was slightly higher than order 2 (Figure 1C). Then, the logarithm of slope (LOS) was applied to analyze the digestion rate. As the digestion nearly reached equilibrium at 3 h, the LOS plots were fitted for the digested starch during this period to obtain the more accurate digestion rate coefficients (Figure 1D). As shown in Figure 1D, the digestion process could be divided into two phases with the regression coefficients in each phase ranging from 0.942 to 0.993, indicating that the digestion of starch in the absence and presence of TA for different mixing procedures conformed to the first-order reaction property. In this model, the digestion rate constant in the first phase (*k*_1_) that mainly represents the hydrolysis of rapidly digestible starch [26] followed the order of no inhibition > order 2 > order 3 > order 1 (Table 1). Therefore, at the selected TA concentration in the time course study, the inhibition effects of α-amylase for the three mixing procedures followed as order 1 > order 3 > order 2.

### 3.2. The Mechanism in α-Amylase Inhibition of TA

**Figure 2:**
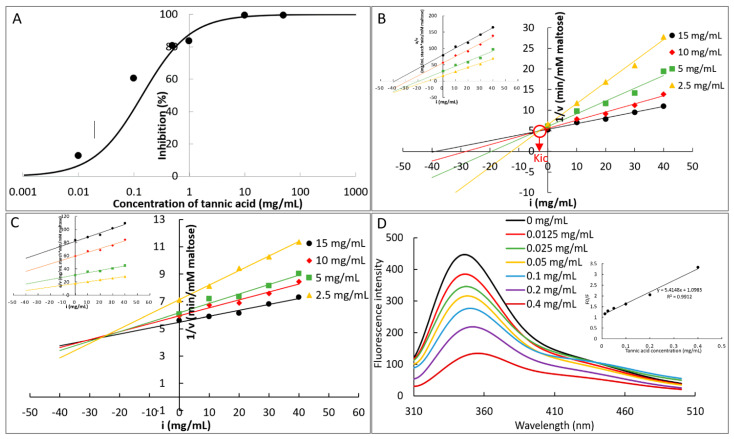
The mechanism in α-amylase inhibition of TA. α-Amylase inhibition at a series of TA concentrations determined by use of an EnzCheck^TM^ ultra amylase assay kit, and the curve was fitted according to the IC_50_ value calculation equation (**A**). Inhibition kinetics of TA for order 2 (**B**) and order 3 (**C**), in which Dixon and Cornish-Bowden (inserted) plots were described to obtain the competitive inhibition constant, *K_ic_*; The quenching effect of TA on α-amylase fluorescence (**D**) and the fluorescence quenching constant *K_FQ_* was obtained from Stern-Volmer equation (inserted).

To describe how TA developed the inhibiting effect against α-amylase, IC_50_ value, inhibition kinetics, and fluorescence quenching were applied. It was found that the enzyme inhibition of TA was dependent on the phenolic concentration (Figure 2A), and the IC_50_ value was calculated as 0.14 mg/mL based on the inhibition (%) at the respective TA concentrations (Figure 2A and Table 1). It should be noted that to obtain the necessary high inhibition ratios at the available TA concentrations for the requirement of IC_50_ value determination, an amylase activity assay kit was applied, in which the expected inhibition effect could be achieved at a relatively lower TA concentration compared to the starch digestion method in Figure S1 (avoiding the insolubility of a large amount of TA). According to the kit manufacturing guideline, only order 1 was applicable for the IC_50_ value measurement of TA (Table 1). Two kinetics equations, including Dixon and Cornish-Bowden equations, which plot the correlations between the initial reaction velocity, inhibitor concentration, and substrate concentration were applied to study the detail inhibition kinetics of TA. It was found that for order 1, the Dixon plots intersected at one point (although there was experimental deviation), while the Cornish-Bowden plots paralleled with each other (Figure 2B), indicating that TA was a competitive inhibitor of α-amylase [22], in accordance with previous studies although the enzyme types are kind of different [16]. The competitive inhibition constant (*K_ic_*) that describes the dissociation of TA-amylase binary complex [16] was obtained from the abscissa of the intersection point in Dixon plot as 3.454 mg/mL (Figure 2B and Table 1). Although the *K_ic_* value for order 2 (25.912 mg/mL) was significantly higher than order 1 (Table 1), the inhibition type was not changed by altering the mixing order (Figure 2B,C). As there was a large variation of the initial reaction velocity at different TA/starch mass ratios for order 3 (data not shown), the kinetics determination could not be fitted with both Dixon and Cornish-Bowden equations. Furthermore, the binding interactions between TA and α-amylase was characterized by fluorescence quenching (Figure 2D), from which the fluorescence quenching constant, *K_FQ_* (9.209 × 10^3^ M^−1^), and the bimolecular quenching constant, *k_q_* (3.1 × 10^12^ M^−1^s^−1^), were obtained according to the Stern-Volmer equation (Table 1). Because the Stern-Volmer plot of TA was a linear type (Figure 2D), the quenching followed only one mechanism, i.e., static (formation of enzyme-quencher complex) or dynamic (random collision) [27]. For the typical dynamic one, the *k_q_* value is around 1 × 10^10^ M^−1^s^−1^ [23,27]. As the *k_q_* value of TA was 300 times higher than this (Table 1), the quenching effect of TA on α-amylase fluorescence arose from the formation of the complex between TA and the enzyme.

### 3.3. Adsorption of TA with Starch

**Figure 3:**
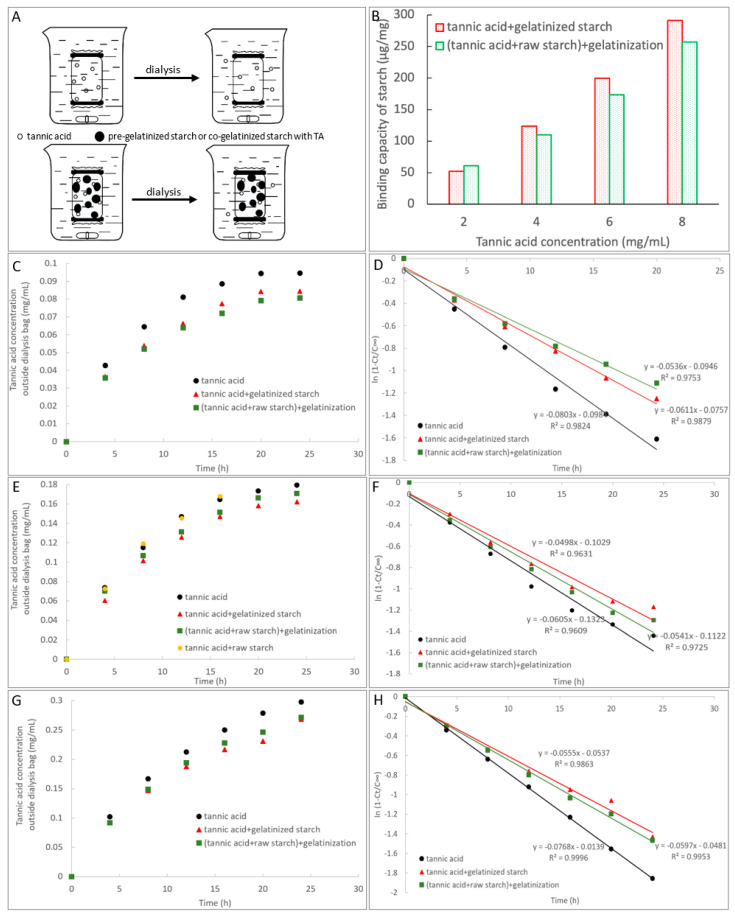
The dialysis scheme of TA for the control (the upper one) and for the TA-starch gelatinized systems (the below one, including two mixing procedures, i.e., mixing TA with pre-gelatinized starch, and co-gelatinizing TA with starch) (**A**). The adsorption capacity (adsorption amount of TA per mass of starch) of starch for two mixing procedures at the dialysis time of 20 h at the low (2 mg/mL) and high (4, 6, and 8 mg/mL) initial TA concentrations in the dialysis bag (**B**). The concentrations of dialyzed TA outside the dialysis bag along with time at 2 (**C**), 4 (**E**), and 6 (**G**) mg/mL of initial TA concentrations inside the bag for two mixing procedures; The logarithm of slope analysis for the fraction of dialyzed TA along with time at 2 (**D**), 4 (**F**), and 6 (**H**) mg/mL of initial TA concentrations inside the bag, from which the transport rate constants *k_t_* that reflect the dialysis velocity were obtained.

To study the adsorption property of TA with starch for two gelatinization procedures, the dialysis of TA in the presence and absence of gelatinized starch were performed (Figure 3A). It was found that the dialysis of TA nearly reached equilibrium at 20 h. Therefore, the adsorption capacity of starch for the two procedures were compared at various TA concentrations (i.e., various TA/starch mass ratios) at this time point (Figure 3B). As shown, at the low TA concentration (2 mg/mL), the adsorption capacity of starch that was co-gelatinized with TA was higher than starch that was pre-gelatinized before TA addition, while at the high TA concentrations (4, 6, and 8 mg/mL), the adsorption capacity was shown with the opposite comparison result (pre-gelatinized > co-gelatinized) (Figure 3B). Then, the time course of TA dialysis was recorded and analyzed by use of first-order kinetics (Figure 3C–H). In the beginning, the dialysis velocity was high, and with the increase in TA concentration outside the dialysis bag, the velocity decreased gradually (Figure 3C,E,G). In addition, the plot of the amount of dialyzed TA against time accorded with the first-order kinetics equation in a good manner during 24 h, from which the transport rate constants *k_t_* were obtained (Figure 3D,F,H). The *k_t_* value in the absence of starch was always the highest at each TA concentrations (Table 2), because nothing hindered the free diffusion of TA. Both gelatinization procedures decreased the *k_t_* value of TA, and the orders in *k_t_* values for the two procedures at each TA concentrations were contrary to the orders in the adsorption capacity of starch (Table 2). Moreover, the raw starch (the ordered branches of starch chains are folded) hardly affected the diffusion of TA inside to outside the dialysis bag in 15 h (Figure 3E), indicating that only the unfolded starch branches during the gelatinization process could significantly show the adsorption effect on TA.

### 3.4. Binding Interactions between TA and Starch

**Figure 4:**
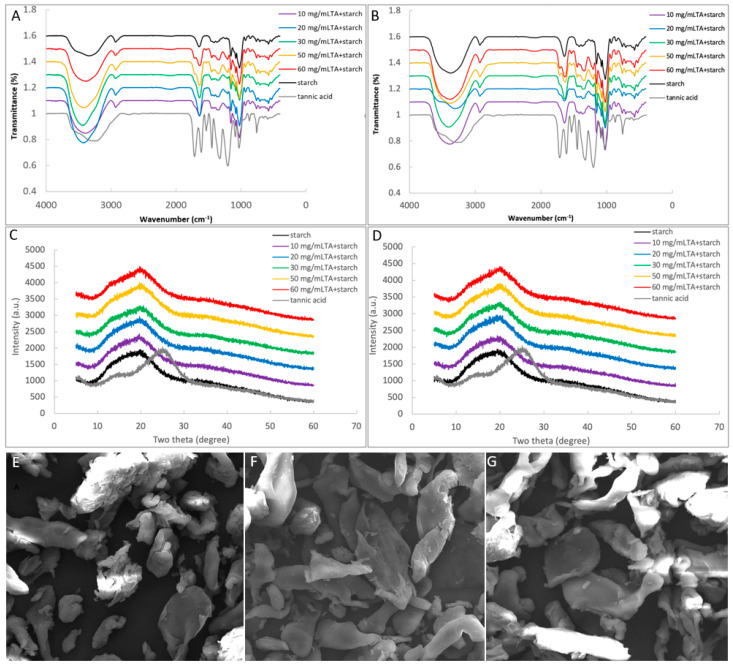
The FTIR spectra of lyophilized gelatinized starch in the absence and presence of TA for mixing TA with pre-gelatinized starch (**A**), and co-gelatinizing TA with starch (**B**). The XRD profiles of lyophilized gelatinized starch in the absence and presence of TA for mixing TA with pre-gelatinized starch (**C**), and co-gelatinizing TA with starch (**D**). The SEM profiles of lyophilized gelatinized starch in the absence (**E**) and presence of TA for mixing TA with pre-gelatinized starch (**F**), and co-gelatinizing TA with starch (**G**) at the magnification of 4000 times.

To study the effect of TA adsorption on the molecular structures of lyophilized starch, the characterizing methods of FTIR, XRD, and SEM were applied, from which the binding interactions between TA and starch could be also reflected. In the FTIR spectra, all the samples were shown with a broad band at 3000–4000 cm^−1^ (Figure 4A,B) that indicates the vibrational stretching of hydroxyl groups [26]. The peaks at 2925 cm^−1^ and 1646 cm^−1^ suggested the stretching vibrations of C-H and C=O groups, respectively. The stretching of C-O (in C-O-H) in the anhydroglucose ring mainly caused the peak at 1080 cm^−1^ [28]. Besides, with the TA addition amount increasing, the peak at 1715 cm^−1^ that represents the stretching vibration of C=O (in O-C=O of TA) [29] became more obvious in the lyophilized TA-starch (Figure 4A,B), indicating the introduction of TA in samples. It was found that all the samples of two gelatinization procedures were shown with similar characteristic peaks to the control: without new peaks, peak shift, and significant increase in peak width at half height of the band at 3000–4000 cm^−1^ (Figure 4A,B). In addition, the ratio of peak height at 1047 cm^−1^ to 1022 cm^−1^ that reflects the degree of short-range order at the surface of starch granules [28] was not significantly altered by TA addition for two gelatinization procedures (Table 2). Due to the gelatinization process applied during which the ordered crystalline structures were completely destroyed, there was no obvious characteristic peaks of the freeze-drying samples in the XRD profiles (Figure 4C,D), with the relative crystallinity of around 16% in the absence of TA (Table 2). TA addition did not significantly change the crystallinity of the gelatinized starch for both gelatinization procedures, but only caused a slight decrease in the crystallinity (Table 2) because of the introduction of TA (this was suggested by the 1715 cm^−1^ peak in the FTIR spectra, Figure 4A,B) that had a lower relative crystallinity (12.62%). In addition, the lyophilized starch samples in the absence and presence TA were all observed by SEM as irregular lamellar and/or large granular characteristics (Figure 4E–G).

## 4. Discussion

**Figure 5:**
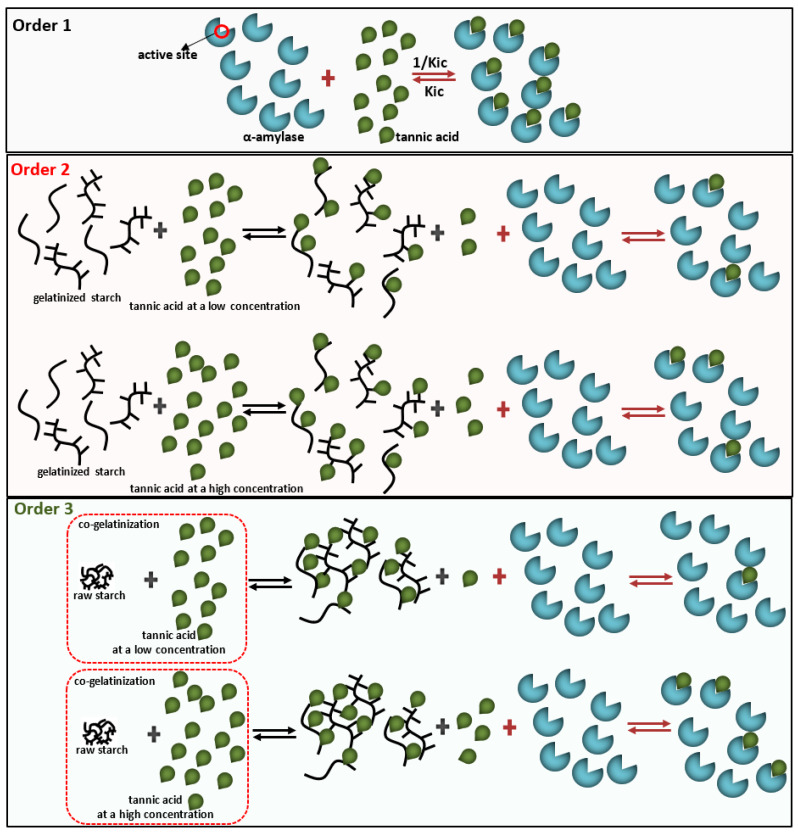
The scheme of effects of mixing orders (procedures) on TA-amylase binding interactions. For order 1, TA was firstly mixed with α-amylase before starch addition; therefore, TA could bind with the active site (in a competitive inhibition manner) of the enzyme thoroughly. Notably, in the binding equilibrium, *K_ic_* indicates the dissociation constant of TA-amylase complex (reforming individual TA and α-amylase); therefore, 1/*K_ic_* suggests the binding constant of TA with the enzyme active site. For order 2, TA was firstly mixed with the pre-gelatinized starch before α-amylase addition. In this order, TA molecules were adsorbed onto the unfolded starch chains in a disordered manner, decreasing the amount of free TA molecules that could bind with α-amylase. Because the inhibition kinetics of TA for this order could still be well-fitted with the competitive Dixon and Cornish-Bowden equations that were performed at a series of starch concentrations (also a series of mass ratios of TA to starch), the adsorption of TA with pre-gelatinized starch at the low and high TA concentrations were suggested to have a similar property. For order 3, TA was mixed with raw starch and then co-gelatinized before α-amylase addition. In this order, TA had a longer contacting process and a higher interacting temperature (than order 2) with starch from the beginning of gelatinization process. Therefore, TA molecules interacted with starch chains more thoroughly (than order 2) along with the swelling of starch granules and unfolding of ordered structures, tending to form a network where TA(s) are included inside acting as a ‘bridge’ linkage of swollen starch. This decreased the binding of TA with α-amylase and the decreasing effect was higher than order 2, specially at a low TA concentration. However, with the TA concentration increasing, the incorporated TA in the network gradually reached saturation, and the entering of additional TA was retarded. By this way, the amount of unbound TA that could bind with α-amylase for this order was more than that for order 2 at a high TA concentration.

The inhibition of α-amylase by a polyphenol has been considered to be caused by binding interactions between them [16]. As one biomacromolecule, the enzyme substrate starch is also able to bind/adsorb with the biomicromolecule polyphenol [11,12,13]. Therefore, the binding of polyphenol with starch may affect the enzyme inhibition effect of the polyphenol. Different performing procedures in α-amylase inhibition in vitro is supposed to cause different binding properties between polyphenol, the enzyme, and the substrate. Tannic acid (TA) is a typical competitive inhibitor that can bind with the active site of α-amylase [16], and thus it is considered as a preferential phenolic compound in studying the characters of competitive inhibition that is the most common inhibition type. Therefore, the effect of mixing orders on α-amylase inhibition of tannic acid was explored in this study. The inhibition of a polyphenol is commonly characterized by the approach that mixes the compound with α-amylase followed by substrate addition (order 1). It was found that both the other conducting approaches that mixing TA with starch before α-amylase addition (order 2 and 3) decreased the inhibition effect of TA (Figure 1B). Interestingly, at the low TA concentration (10 mg/mL) the inhibition of TA that was co-gelatinized with starch (order 3) was lower than TA that was mixed with pre-gelatinized starch (order 2), while at the high TA concentrations (20 and 30 mg/mL) the inhibition effects were shown with the opposite trends (order 3 > order 2) (Figure 1B). Therefore, the binding interactions between TA and starch are supposed to vary with the processing methods and the mass ratios (TA/starch). As a result, the amounts of free (unbound) TA that can develop the inhibitory activity against the enzyme were different. Time course of starch digestion in the absence and presence of TA for three mixing procedures were drawn to describe the inhibition effects, from which the digestion rate constants (*k*) were obtained to reflect the catalytic ability of α-amylase, especially for the first phase as the substrate (rapidly digestible starch) is able to bind with the enzyme efficiently and thus can be catalyzed efficiently in this stage [28]. Therefore, a higher value of *k*_1_ (digestion rate constant in the first phase) suggests a higher enzymic ability and thus a lower inhibitory activity [19]. During the whole digestion course studied, TA that was firstly mixed with α-amylase always showed the highest inhibitory activity as suggested by the lowest *k*_1_ value of order 1 (Figure 1D and Table 1). This arises from the fact that in this procedure, TA has more opportunity to contact/bind with α-amylase directly compared to order 2 and 3, causing better competitive effect with starch in terms of binding with the active site of the enzyme (Figure 5). Notably, although TA caused the inhibition of starch digestion for order 2 and 3, the final ratios of digestible starch for both orders were similar to that for no inhibition (Figure 1C). This indicates that the binding interactions of TA with starch did not significantly alter the molecular conformation and/or gelatinization degree of the gelatinized starch; otherwise, the digestible ratio would be changed, as starch configuration and gelatinization degree are two main factors deciding the digestibility [13,26,30].

In the inhibition kinetics study, the competitive inhibition constant *K_ic_* describes the dissociation of TA-amylase by definition; therefore, the reciprocal of competitive inhibition constant, 1/*K_ic_*, indicates the binding affinity of TA to the enzyme active site (Figure 5) [22]. Through this, mixing TA with gelatinized starch (order 2) was suggested to decrease the binding of TA with α-amylase, because the 1/*K_ic_* value of order 2 was lower than that of order 1 (Table 1). This resulted in the decreased inhibition effect of TA for order 2 compared to order 1, as indicated by the inhibition percentage at each TA concentrations (order 2 < order 1) and the digestion rate constant (*k*_1_ value, order 2 > order 1) (Figure 1B and Table 1). Notably, order 2 only reduced the inhibition effect but with the inhibition type untouched (Figure 2B,C), indicating that the binding of TA with pre-gelatinized starch were shown with the similar adsorption property at different mass ratios of TA to starch (otherwise the inhibition kinetics determination in order 2 may not be fitted well with the competitive Dixon and Cornish-Bowden plots). On the other hand, the inhibition kinetics determination in order 3 could not be fitted suitably, which is also suggested by the large gap between the inhibition at the low and high TA concentrations in order 3 (Figure 1B). This indicates that the binding of TA with starch during the co-gelatinization process varied with the mass ratios of TA to starch. Specifically, the binding/adsorption between TA and starch in different procedures are discussed as follows.

To describe the property of adsorption of TA to starch in solution, a dialysis method was applied by which a mixture of TA and gelatinized starch or a solution of co-gelatinized TA-starch were kept in a dialysis bag, respectively (Figure 3A). The regression efficiency (*R*^2^) of the fitting model applied for diffusion of TA inside the dialysis bag to outside in the absence and presence of TA were all above 0.96 (Figure 3D,F,H), indicating that the transport of TA conformed to first-order kinetics of pharmacodynamics. In this kinetics model, a higher value of transport rate constant *k_t_* indicates a higher diffusion velocity of TA. Therefore, gelatinized starch was suggested to bind with TA, limiting the free diffusion of the phenolic micromolecule, indicated by the lower *k_t_* values in the presence of gelatinized starch (both pre-gelatinization before TA addition and co-gelatinization with TA) than that in the absence of starch (Table 2). Thus, the decreased inhibitory activity of TA against α-amylase for order 2 and 3 are proved to result from the binding of TA of gelatinized starch that occurred before α-amylase addition and limited the binding of free TA with the enzyme. It should be noted that at the low TA concentration the *k_t_* value of TA that was co-gelatinized with starch was lower than TA that was mixed with pre-gelatinized starch (Table 2), suggesting that the co-gelatinization processing promoted the TA-starch binding interactions at such TA/starch mass ratio. This may be caused by the fact that TA had a longer contacting process and a higher interacting temperature (than order 2) with starch from the beginning of gelatinization process. Therefore, TA molecules interacted with starch chains more thoroughly (than order 2) along with the swelling of starch granules and the unfolding of microcrystalline structural parts, tending to form a network (although it seems impact) where TA(s) were assembled inside the network acting as a ‘bridge’ linkage of swollen starch (Figure 5) [31]. As a result, a higher amount of TA was absorbed into the starch after co-gelatinization than when the TA is mixed only with pre-gelatinized starch (where TA tended to be adsorbed onto starch in a disordered form, as the pre-gelatinized starch is arrayed in total disorder) (Figure 5). Therefore, the amount of free TA in the gelatinized system that can develop the inhibitory effect in order 3 was less than that in order 2 (Figure 5). On the other hand, at a high TA concentration, the *k_t_* values were shown with the opposite trend (pre-gelatinization before TA addition < co-gelatinization with TA) (Table 2), indicating that pre-gelatinized starch tends to bind more TA in the same dialysis duration. In the co-gelatinization system, as with the concentration of TA increasing, the incorporated TA in the network as discussed above gradually reached saturation, and the entering of additional TA was limited (or retarded). In the meanwhile, in the pre-gelatinized system the unfolded starch branches could still adsorb the increasing amount of TA due to a disordered absorbing interaction (Figure 5). This is supported by the higher binding capacity of pre-gelatinized starch relative to the co-gelatinized one at the high TA concentrations (Figure 3B). Therefore, at a high TA concentration more free TA exists in the co-gelatinization mixture (Figure 5), causing the higher inhibitory effect for order 3 than order 2 (Figure 1B).

Furthermore, to illustrate the effect of TA binding on the microcrystalline structures and apparent morphology of starch, FTIR, XRD, and SEM were applied in characterization of lyophilized gelatinized starch in the presence and absence of TA. As there were no new peaks observed in TA-starch complexes (both for pre-gelatinization before TA addition and co-gelatinization with TA), compared to the respective FTIR spectra of TA and starch (Figure 4A,B), the binding of TA with starch resulted from non-covalent physical absorption. In addition, neither significant redshift nor increase in full width at half height of peaks at 3000–3500 cm^−1^ (vibrational stretching of both inter- and intra-molecular hydroxyl groups) were observed for all the TA-starch samples (Figure 4A,B), suggesting that the binding interactions between TA and starch may not be mainly attributed to hydrogen bondings [28,32]. This is not surprising as the treatments in this study (both mixing and co-gelatinization) are considered relatively gentle compared to some field forces, like microwave, ultrasonic, plasma, etc. [28,33], under which hydrogen bondings formed between phenolic compounds and starch molecules due to the enhanced intermolecular collision [34,35]. Instead, the physical absorption of TA to starch during the mixing and co-gelatinization processes may arise from other weaker molecular interactions, e.g., Van der Waal’s force, which widely exists in two or more molecules [32,36]. In addition, the degree of short-range order of the starch microcrystalline region (as suggested by the ratio of absorbance at peak 1047 cm^−1^ to 1022 cm^−1^) [37] was not changed by TA addition (Table 2), which is in accordance with the untouched relative crystallinities of starch bound with TA as suggested by XRD (Table 2). The digestibility of one kind of starch is dependent on its molecular structures, of which the microcrystalline degree is one important factor as it decides the unfolding extent of the ordered starch chain region and the binding with α-amylase [38]. Therefore, although the reaction velocity of TA-starch digestion for order 2 was higher than that for order 3, especially in the first digestion phase (Figure 1D and Table 1), the final percentages of digestible starch in both orders were even similar to that without TA addition due to the untouched (micro-)crystalline structures of starch in the presence of TA. Notably, it is not surprising that although the binding affinity of TA to starch was different between the pre-gelatinization and co-gelatinization processing (Figure 3). TA addition remained the relative crystalline degree of lyophilized starch (Table 2), because during freeze-drying retrogradation the intra- and inter-molecular interactions of starch chains themselves were much stronger than the weak interactions between TA and starch [39]. Moreover, the apparent morphology of lyophilized starch was not altered by TA (Figure 4E–G). Therefore, taking both the microscopic and macroscopic structures into account, the addition of TA before and after starch gelatinization at the applied mass ratios of TA/starch may not affect the processing properties of starch but is able to delay the digestion velocity potentially through the inhibition effect on α-amylase.

The three orders in this study correspond to three supplementary modes of polyphenols in diets, e.g., (1) intaking polyphenols, phenolic extracts, or foods rich in polyphenols before meals (order 1). In this case, polyphenols have more opportunity for binding with α-amylase, developing the inhibitory activity and delaying digestion of starch in meal; (2) intaking polyphenols (extracts or foods) together with meal. In this case, the phenolic compounds could be adsorbed onto the gelatinized starch in meal, which affects the binding of polyphenols with α-amylase and thus decreases the inhibition effects; (3) starchy foods are processed together with polyphenols for healthy food production. In this case, the different polyphenol/starch mass ratios may cause different polyphenol binding capacity of starch, and this further affects the amounts of unbound polyphenols that have the inhibitory activity against α-amylase. Notably, the addition of polyphenols discussed here did not significantly change the microcrystalline and integral morphology of lyophilized starch due to the relatively weak interactions between them. However, for the polyphenols that are able to interact with starch molecules under some certain external filed forces through relatively strong non-covalent forces, like hydrogen bonding and hydrophobic force, the changes in starch digestibility and digestion velocity may be attributed to the increase in relative crystallinity of starch [28,40]. Furthermore, although adding polyphenols into the mixture of α-amylase and starch was not studied, the inhibitory activity for this order is considered as the lowest because the enzyme preferentially binds with and catalyzes substrates [16]. Conclusively, when evaluating the inhibitory activity of a polyphenol, the effect of the mixing order of inhibitor (polyphenol), substrate (starch), and enzyme (α-amylase) is suggested to be taken into account as the binding of polyphenol with α-amylase may be affected by the adsorption of polyphenol with starch in the case that the polyphenol contacts with starch earlier than with the enzyme.

## 5. Conclusions

By taking three common processing procedures of polyphenols, starch, and α-amylase in actual case into account, the effects of mixing orders on the inhibitory activity of tannic acid against α-amylase were explored in this study. Mixing TA with α-amylase before starch addition caused the highest inhibiting effects at each TA concentrations because TA had more opportunity for contacting and binding with α-amylase before the enzyme specifically bound with and catalyzed starch. As for two procedures where mixing TA with starch before α-amylase addition (mixing TA with pre-gelatinized starch, and co-gelatinizing starch with TA), gelatinized starch could adsorb TA through weak non-covalent interactions like Van der Waal’s force, which decreased the binding of TA with the active site of α-amylase. However, the adsorption capacity of the pre-gelatinized starch was lower than the co-gelatinized starch at a low TA concentration, and higher than the co-gelatinized starch at a high TA concentration, which may result from the different TA-starch adsorption mechanisms for the two gelatinization procedures. This resulted in the difference in the inhibitory effect of unbound TA. To evaluate the inhibitory activity of a phenolic compound in diets, in addition to molecular structure of the polyphenol and other existing components, supplementary modes of the polyphenol may be also considered because different mixing orders of the inhibitor, enzyme, and substrate are supposed to cause different inhibition effects as suggested in this study.

## Figures and Tables

**Table 1:** The methods and corresponding constants that characterize α-amylase inhibition of TA for different mixing orders of the inhibitor, enzyme, and substrate.

Methods	Mixing Orders	Constants Characterizing α-Amylase Inhibition of TA	
IC_50_ value	(TA + α-amylase) + ‘DQ starch’	IC_50_ value (mg/mL)	0.140
Inhibition effect	Order 1	Inhibition (10, 20, and 30 mg/mL TA, %)	35.82 ^a^, 56.06 ^a^, and 67.67 ^a^
	Order 2		7.58 ^b^, 24.76 ^c^, and 36.87 ^c^
	Order 3		1.56 ^c^, 29.45 ^b^, and 58.18 ^b^
Logarithm of slope (LOS) analysis	No inhibition	Digestion rate constant (*k*_1_ and *k*_2_, min^−1^)	0.0180 ^a^ and 0.0138 ^a^
	Order 1		0.0088 ^d^ and 0.0039 ^c^
	Order 2		0.0125 ^b^ and 0.0064 ^b^
	Order 3		0.0119 ^bc^ and 0.0068 ^b^
Inhibition kinetics	Order 1	Competitive inhibition constant and its reciprocal (*K_ic_* and 1/*K_ic_*, mg/mL and mL/mg)	3.454 ^b^ and 0.290 ^a^
	Order 2		25.912 ^a^ and 0.038 ^b^
Fluorescence quenching	TA + α-amylase	Fluorescence quenching constant *(K_FQ_*, M^−1^) Bimolecular quenching constant (M^−1^s^−1^)	9.209 × 10^3^ 3.1 × 10^12^

Note: For the methods of inhibition effect, LOS analysis, and inhibition kinetics, the different superscript letters in the same column indicate the constants are significantly different (*p* < 0.05) with other for different orders. As for the methods of IC_50_ value and fluorescence quenching, they are only applied for order 1 (mixing TA with α-amylase firstly) due to the requirement of the respective determination approach.

**Table 2:** The methods and corresponding constants that characterize the binding/adsorption of TA with starch for two gelatinization procedures.

	Gelatinization Procedures	Constants Characterizing Binding/Adsorption of TA with Starch			
Dialysis	TA	Transport rate constant (*k_t_*, min^−1^)	0.0803 ^a^ (2 mg/mL TA)	0.0605 ^a^ (4 mg/mL TA)	0.0768 ^a^ (6 mg/mL TA)
	TA + gelatinized starch		0.0611 ^b^ (2 mg/mL TA)	0.0498 ^c^ (4 mg/mL TA)	0.0555 ^c^ (6 mg/mL TA)
	(TA + raw starch) + gelatinization		0.0536 ^c^ (2 mg/mL TA)	0.0541 ^b^ (4 mg/mL TA)	0.0597 ^b^ (6 mg/mL TA)
FTIR	Gelatinized starch	*R*-1047 cm^−1^/1022 cm^−1^	0.797 ^a^ (control for TA + gelatinized starch), 0.745 ^a^ (control for (TA + raw starch) + gelatinization)		
	TA + gelatinized starch		0.757 ^ab^ (10 mg/mL TA)	0.804 ^a^ (30 mg/mL TA)	0.774 ^a^ (50 mg/mL TA)
	(TA + raw starch) + gelatinization		0.731 ^a^ (10 mg/mL TA)	0.727 ^a^ (50 mg/mL TA)	0.727 ^a^ (60 mg/mL TA)
XRD	Gelatinized starch	Crystallinity (%)	16.32 ^a^ (control for TA + gelatinized starch), 16.25 ^a^ (control for (TA + raw starch) + gelatinization)		
	TA + gelatinized starch		16.17 ^a^ (10 mg/mL TA)	15.76 ^ab^ (30 mg/mL TA)	15.16 ^ab^ (50 mg/mL TA)
	(TA + raw starch) + gelatinization		15.95 ^a^ (10 mg/mL TA)	15.67 ^a^ (30 mg/mL TA)	15.39 ^a^ (50 mg/mL TA)

Note: For the method of dialysis, the different superscript letters in the same column indicate that the *k_t_* values are significantly different (*p* < 0.05) with each other at the respective TA concentrations. For the methods of FTIR and XRD, the values of *R*-1047 cm^−1^/1022 cm^−1^ and relative crystallinity are analyzed regarding the significant difference between the respective mixing procedure and its corresponding control.

## Data Availability

Not applicable.

## Supplementary Materials

The following are available online at https://www.mdpi.com/article/10.3390/foods10061233/s1, Figure S1: Three mixing orders (procedures) of tannic acid (TA), starch and α-amylase applied in this study.
