# Supplementary material for: The Physical Adsorption of Gelatinized Starch with Tannic Acid Decreases the Inhibitory Activity of the Polyphenol against α-Amylase
Source: Foods. 2021 May 28;10(6):1233. doi: 10.3390/foods10061233 (PMC8226663; doi:10.3390/foods10061233)
Supplement: Supplementary file 1 [file foods-10-01233-s001.zip › foods-1157271-supplementary.pdf]

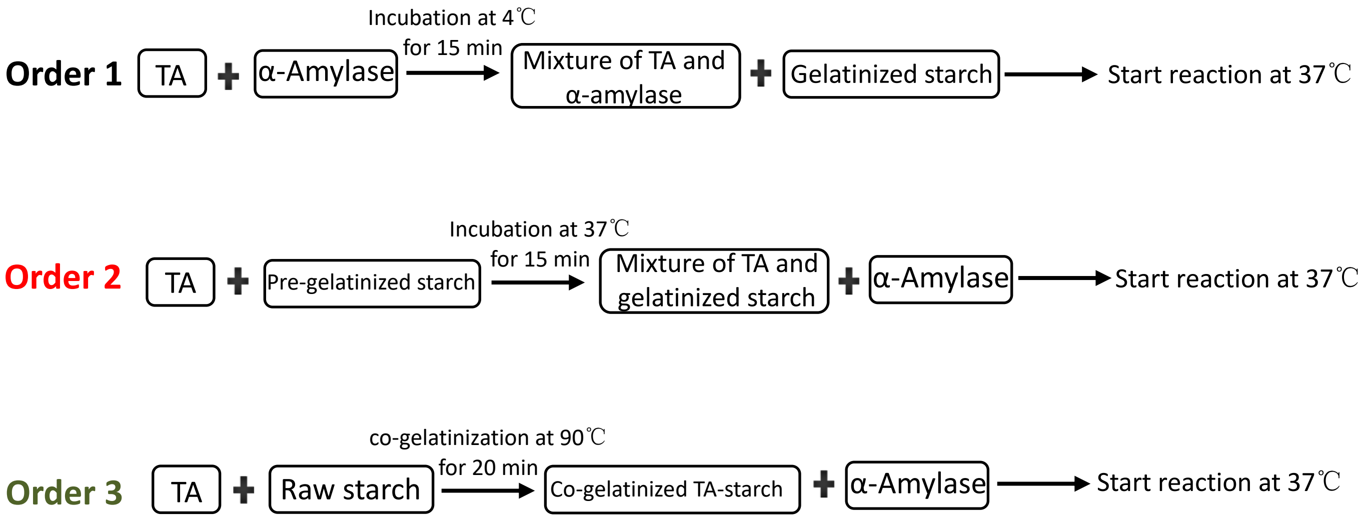
Order 1
TA
α-Amylase
Incubation at 4℃
for 15 min
Mixture of TA and α-amylase
Gelatinized starch
Start reaction at 37℃
Order 2
TA
Pre-gelatinized starch
Incubation at 37℃
for 15 min
Mixture of TA and gelatinized starch
α-Amylase
Start reaction at 37℃
Order 3
TA
Raw starch
co-gelatinization at 90℃
for 20 min
Co-gelatinized TA-starch
α-Amylase
Start reaction at 37℃
